# Using the Relevance Vector Machine Model Combined with Local Phase Quantization to Predict Protein-Protein Interactions from Protein Sequences

**DOI:** 10.1155/2016/4783801

**Published:** 2016-05-23

**Authors:** Ji-Yong An, Fan-Rong Meng, Zhu-Hong You, Yu-Hong Fang, Yu-Jun Zhao, Ming Zhang

**Affiliations:** ^1^School of Computer Science and Technology, China University of Mining and Technology, Xuzhou, Jiangsu 21116, China; ^2^Key Laboratory of Network Oriented Intelligent Computation, Harbin Institute of Technology Shenzhen Graduate School, Shenzhen, Guangdong 518055, China

## Abstract

We propose a novel computational method known as RVM-LPQ that combines the Relevance Vector Machine (RVM) model and Local Phase Quantization (LPQ) to predict PPIs from protein sequences. The main improvements are the results of representing protein sequences using the LPQ feature representation on a Position Specific Scoring Matrix (PSSM), reducing the influence of noise using a Principal Component Analysis (PCA), and using a Relevance Vector Machine (RVM) based classifier. We perform 5-fold cross-validation experiments on* Yeast* and* Human* datasets, and we achieve very high accuracies of 92.65% and 97.62%, respectively, which is significantly better than previous works. To further evaluate the proposed method, we compare it with the state-of-the-art support vector machine (SVM) classifier on the* Yeast* dataset. The experimental results demonstrate that our RVM-LPQ method is obviously better than the SVM-based method. The promising experimental results show the efficiency and simplicity of the proposed method, which can be an automatic decision support tool for future proteomics research.

## 1. Introduction

Proteins are crucial molecules that participate in many cellular functions in an organism. Typically, proteins do not perform their roles individually, so detection of PPIs becomes more and more important. Knowledge of PPIs can provide insight into the molecular mechanisms of biological processes and lead to a better understanding of practical medical applications. In recent years, various high-throughput technologies, such as yeast two-hybrid screening methods [1, 2], immunoprecipitation [3], and protein chips [4], have been developed to detect interactions between proteins. Until now, a large quantity of PPI data for different organisms has been generated, and many databases, such as MINT [5], BIND [6], and DIP [7], have been built to store protein interaction data. However, these experimental methods have some shortcomings, such as being time-intensive and costly. In addition, the aforementioned approaches suffer from high rates of false positives and false negatives. For these reasons, predicting unknown PPIs is considered a difficult task using only biological experimental methods.

As a result, a number of computational methods have been proposed to infer PPIs from different sources of information, including phylogenetic profiles, tertiary structures, protein domains, and secondary structures [8–16]. However, these approaches cannot be employed when prior knowledge about a protein of interest is not available. With the rapid growth of protein sequence data, the protein sequence-based method is becoming the most widely used tool for predicting PPIs. Consequently, a number of protein sequence-based methods have been developed for predicting PPIs. For example, Bock and Gough [17] used a support vector machine (SVM) combined with several structural and physiochemical descriptors to predict PPIs. Shen et al. [18] developed a conjoint triad method to infer human PPIs. Martin et al. [19] used a descriptor called the signature product of subsequences and an expansion of the signature descriptor based on the available chemical information to predict PPIs. Guo et al. [20] used the SVM model combined with an autocorrelation descriptor to predict* Yeast* PPIs. Nanni and Lumini [21] proposed a method based on an ensemble of K-local hyperplane distances to infer PPIs. Several other methods based on protein amino acid sequences have been proposed in previous work [22, 23]. In spite of this, there is still space to improve the accuracy and efficiency of the existing methods.

In this paper, we propose a novel computational method that can be used to predict PPIs using only protein sequence data. The main improvements are the results of representing protein sequences using the LPQ feature representation on a Position Specific Scoring Matrix (PSSM), reducing the influence of noise by using a Principal Component Analysis (PCA), and using a Relevance Vector Machine (RVM) based classifier. More specifically, we first represent each protein using a PSSM representation. Then, a LPQ descriptor is employed to capture useful information from each protein PSSM and generate a 256-dimensional feature vector. Next, dimensionality reduction method PCA is used to reduce the dimensions of the LPQ vector and the influence of noise. Finally, the RVM model is employed as the machine learning approach to carry out classification. The proposed method was executed using two different PPIs datasets:* Yeast* and* Human*. The experimental results are found to be superior to SVM and other previous methods, which prove that the proposed method performs incredibly well in predicting PPIs.

## 2. Materials and Methodology

### 2.1. Dataset

To verify the proposed method, two publicly available datasets are used in our study. The datasets are* Yeast* and* Human* that were obtained from the publicly available Database of Interaction Proteins (DIP) [24]. For better implementation, we selected 5594 positive protein pairs to build the positive dataset and 5594 negative protein pairs to build the negative dataset from the* Yeast* dataset. Similarly, we selected 3899 positive protein pairs to build the positive dataset and 4262 negative protein pairs to build the negative dataset from the* Human* dataset. Consequently, the* Yeast* dataset contains 11188 protein pairs and the* Human* dataset contains 8161 protein pairs.

### 2.2. Position Specific Scoring Matrix

A Position Specific Scoring Matrix (PSSM) is an *M* × 20 matrix *X* = {*X*_*ij*_:  *i* = 1 ⋯ *M*, *j* = 1 ⋯ 20} for a given protein, where *M* is the length of the protein sequence and 20 represents the 20 amino acids [28–33]. A score *X*_*ij*_ is allocated for the *j*th amino acid in the *i*th position of the given protein sequence in the PSSM. The score *X*_*ij*_ of the position of a given sequence is expressed as *X*_*ij*_ = ∑_*k*=1_^20^ *p*(*i*, *k*) × *q*(*j*, *k*), where *p*(*i*, *k*) is the ratio of the frequency of the *k*th amino acid appearing at position *i* of the probe to be the total number of probes and *q*(*j*, *k*) is the value of Dayhoff's mutation matrix [34] between the *j*th and *k*th amino acids [35–37]. As a result, a high score represents a largely conserved position and a low score represents a weakly conserved position [38–40].

PSSMs are used to predict protein folding patterns, protein quaternary structural attributes, and disulfide connectivity [41, 42]. Here, we also use PSSMs to predict PPIs. In this paper, we used the Position Specific Iterated BLAST (PSI-BLAST) [43] to create PSSMs for each protein sequence. The *e*-value parameter was set as 0.001, and three iterations were selected for obtaining broadly and highly homologous sequences in the proposed method. The resulting PSSMs can be represented as 20-dimensional matrices. Each matrix is composed of *L* × 20 elements, where *L* is the total number of residues in a protein. The rows of the matrix represent the protein residues, and the columns of the matrix represent the 20 amino acids.

### 2.3. Local Phase Quantization

Local Phase Quantization (LPQ) has been described in detail in the literature [44]. The LPQ method is based on the blur invariance property of the Fourier phase spectrum [45–47]. It is an operator used to process spatial blur in textural features of images. The spatial invariant blurring of an original image *f*(*x*) apparent in an observed image *g*(*x*) can be expressed as a convolution, given by

(1) $$\begin{array}{c} g\left(\;x\;\right)=f\left(\;x\;\right)*h\left(\;x\;\right), \end{array}$$

where *h*(*x*) is the function of the spread point of the blur, *∗* represents two-dimensional convolutions, and *x* is a vector of coordinates [*x*, *y*]^*T*^. In the Fourier domain, this amounts to

(2) $$\begin{array}{c} G\left(\;u\;\right)=F\left(\;u\;\right)\cdot H\left(\;u\;\right), \end{array}$$

where *G*(*u*), *F*(*u*), and *H*(*u*) are the discrete Fourier transforms (DFT) of the blurred image *g*(*x*), the original image *f*(*x*), and *h*(*x*), respectively, and *u* is a vector of coordinates [*u*, *v*]^*T*^. According to the characteristic of the Fourier transform, the phase relations can be expressed as

(3) $$\begin{array}{c} ∠G\left(\;u\;\right)=∠F\left(\;u\;\right)+∠H\left(\;u\;\right). \end{array}$$

When the spread point function *h*(*x*) is the center of symmetry, meaning *h*(*x*) = *h*(−*x*), the Fourier transform of *h*(*x*) always has a real value. As a result, its phase can be expressed as a two-valued function, given by

(4) $$\begin{array}{c} ∠H\left(\;u\;\right)=\left\{\begin{array}{cc} 0 & if\;\;H\left(u\right)\geq 0\\ \pi & if\;\;H\left(u\right)<0. \end{array}\right. \end{array}$$

This means that

(5) $$\begin{array}{c} ∠G\left(\;u\;\right)=∠F\left(\;u\;\right). \end{array}$$

The shape of the point spread function *h*(*x*) is similar to the Gaussian or Sin function. This ensures that *H*(*u*) ≥ 0 and *∠G*(*u*) = *∠F*(*u*) at low frequencies, which means that the phase characteristics are due to blur invariance. The local phase information can be extracted using the two-dimensional DFT in LPQ. In other words, a short-term Fourier transform (STFT) computed over a rectangular *M* × *M* neighborhood *N*_*x*_ at each pixel position *x* of an image *f*(*x*) is represented by

(6) $$\begin{array}{c} F\left(\;u,x\;\right)=\underset{y\in N_{x}}{\sum }{f\left(x-y\right)e}^{-j2\pi {yu}^{T}}=w_{u}^{T}f_{x}, \end{array}$$

where *w*_*u*_ is the basis vector of the two-dimensional DFT at frequency *u* and *f*_*x*_ is another vector containing all *M* ^2^ image samples from *N*_*x*_. Using LPQ, the Fourier coefficients of four frequencies are calculated: *u*_1_ = [*a*, 0]^*T*^, *u*_2_ = [0, *a*]^*T*^, *u*_3_ = [*a*, *a*]^*T*^, and *u*_4_ = [*a*, −*a*]^*T*^, where *a* is a small enough number to satisfy *h*(*u*) ≥ 0. As a result, each pixel point can be expressed as a vector, given by

(7) Fxc=Fu1,x,Fu2,x,Fu3,x,Fu4,x,Fx=Re⁡Fxc,Im⁡FxcT.

Then, using a simple scalar quantizer, the resulting vectors are quantized, given by

(8) $$\begin{array}{c} q_{j}\left(\;x\;\right)=\left\{\begin{array}{cc} 1, & if\;\;g_{j}\left(x\right)\geq 0\\ 0, & otherwise, \end{array}\right. \end{array}$$

where *g*_*j*_(*x*) is the *j*th component of  *F*_*x*_. After quantization, *F*_*x*_ becomes an eight-bit binary number vector, and each component of *F*_*x*_ is assigned a weight of 2^*j*^. As a result, the quantized coefficients are represented as integer values between 0 and 255 using binary coding

(9) $$\begin{array}{c} f_{LPQ}\left(\;x\;\right)=\overset{7}{\underset{0}{\sum }}q_{j}\left(\;x\;\right)2^{j}. \end{array}$$

Finally, a histogram of these integer values from all image positions is composed and used as a 256-dimensional feature vector in classification. In this paper, the PSSM matrixes of each protein from the* Yeast* and* Human* datasets were converted to 256-dimensional feature vectors using this LPQ method.

### 2.4. Principal Component Analysis

Principal Component Analysis (PCA) is widely used to process data and reduce the dimensions of datasets. In this way, high-dimensional information can be projected to a low-dimensional subspace, while retaining the main information. The basic principle of PCA is as follows.

A multivariate dataset can be expressed as the following matrix *X*:

(10) X=x1⋮xN,xt=x1t,…,xst,  t=1,…,N,

where *s* is the number of variables and *N* is the number of samplings of each variable. PCA closely related to singular value decomposition (SVD) of matrix and the singular value decomposition of matrix *X* as follows:

(11) $$\begin{array}{c} X=\overset{s}{\underset{i=1}{\sum }}a_{i}b_{i}c_{i}^{T}, \end{array}$$

where *c*_*i*_ represent feature vector of *X* ^*T*^ *X* and *b*_*i*_ represent feature vector of *XX* ^*T*^ and *a*_*i*_ is singular value. If there are *m* linear relationships between *s* variables, then *m* singular values are zero. Any line of *X* can be expressed as feature vector (*q*_1_, *q*_2_,…, *q*_*k*_):

(12) $$\begin{array}{c} X^{T}\left(\;t\;\right)=\overset{k}{\underset{i=1}{\sum }}a_{i}b_{i}c_{i}=\overset{k}{\underset{i=1}{\sum }}r_{i}\left(\;t\;\right)q_{i}, \end{array}$$

where *r*_*i*_(*t*) = *x*(*t*)*q*_*i*_ is projection *x*(*t*) on *q*_*i*_, feature vector (*q*_1_, *q*_2_,…, *q*_*k*_) is load vector, and *r*_*i*_(*t*) is score.

When there is a certain degree of linear correlation between the variables of matrix, then the projection of final several load vectors of matrix *X* will be enough small for resulting from measurement noise. As a result, the principal decomposition of matrix *X* is represented by

(13) $$\begin{array}{c} X=r_{1}q_{1}^{T}+r_{2}q_{2}^{T}+\cdots +r_{k}q_{k}^{T}+E, \end{array}$$

where *E* is error matrix and can be ignored. This does not bring about the obvious loss of useful information of data. In this paper, for the sake of reducing the influence of noise and improving the prediction accuracy, we reduce the dimensionality of the* Yeast* dataset from 256 to 180 and dimensionality of the* Human* dataset from 256 to 172 in the proposed method by using Principal Component Analysis.

### 2.5. Relevance Vector Machine

The characteristics of the Relevance Vector Machine have been described in detail in the literature [48]. For binary classification problems, assume that the training sample sets are {*x*_*n*_, *t*_*n*_}_*n*=1_^*N*^, *x*_*n*_ ∈ *R* ^*d*^ is the training sample, *t*_*n*_ ∈ {0,1} represents the training sample label, *t*_*i*_ represents the testing sample label, and *t*_*i*_ = *y*_*i*_ + *ε*_*i*_, where *y*_*i*_ = *w* ^*T*^ *φ*(*x*_*i*_) = ∑_*j*=1_^*N*^ *w*_*j*_ *K*(*x*_*i*_, *x*_*j*_) + *w*_0_ is the model of classification prediction; *ε*_*i*_ is additional noise, with a mean value of zero and a variance of *σ* ^2^, where *ε*_*i*_ ~ *N*(0, *σ* ^2^), *t*_*i*_ ~ *N*(*y*_*i*_, *σ* ^2^). Assuming that the training sample sets are independent and identically distributed, the observation of vector *t* obeys the following distribution [49–51]:

(14) $$\begin{array}{c} p\left(\;t\;|\;x,w,\sigma^{2}\;\right)={\left(\;2\pi \sigma^{2}\;\right)}^{-N/2}\exp\left[\;-\frac{1}{2\sigma^{2}}{\left\|\;t-\varphi w\;\right\|}^{2}\;\right], \end{array}$$

where *φ* is defined as follows:

(15) $$\begin{array}{c} \varphi =\left(\begin{array}{cccc} 1 & k\left(x_{1},x_{1}\right) & \cdots & k\left(x_{1},x_{N}\right)\\ \vdots & \vdots & \cdots & \vdots \\ 1 & k\left(x_{N},x_{1}\right) & \ldots & k\left(x_{N},x_{N}\right) \end{array}\right). \end{array}$$

The RVM uses sample label *t* to predict the testing sample label *t*_*∗*_, given by

(16) $$\begin{array}{c} p\left(\;t_{*}\;|\;t\;\right)=\int p\left(\;t_{*}\;|\;w,\sigma^{2}\;\right)p\left(\;w,\sigma^{2}\;|\;t\;\right)dw\;d\sigma^{2}. \end{array}$$

To make the value of most components of the weight vector *w* zero and to reduce the computational work of the kernel function, the weight vector *w* is subjected to additional conditions. Assuming that *w*_*i*_ obeys a distribution with a mean value of zero and a variance of *α*_*i*_^−1^, the mean *w*_*i*_ ~ *N*(0, *α*_*i*_^−1^), *p*(*w*∣*a*) = ∏_*i*=0_^*N*^ *p*(*w*_*i*_∣*a*_*i*_), where *a* is a hyperparameters vector of the prior distribution of the weight vector *w*. Hence,

(17) pt∗ ∣ t=∫pt∗ ∣ w,a,σ2pw,a,σ2 ∣ tdw da dσ2,pt∗ ∣ w,a,σ2=Nt∗ ∣ yx∗;w,σ2.

Because *p*(*w*, *a*, *σ* ^2^∣*t*) cannot be obtained by an integral, it must be resolved using a Bayesian formula, given by

(18) pw,a,σ2 ∣ t=pw ∣ a,σ2,tpa,σ2 ∣ t,pw ∣ a,σ2,t=pt ∣ w,σ2pw ∣ apt ∣ a,σ2.

The integral of the product of *p*(*t*∣*a*, *σ* ^2^) and *p*(*w*∣*a*) is given by

(19) pt ∣ a,σ2=2π−N/2Ω−1/2exp⁡−tTΩ−1t2,Ω=σ2I+φA−1φT,A=diag⁡a0,a1,…,aN,pw ∣ a,σ2,t=2π−N+1/2Σ−1/2exp⁡−w−uTw−u2,Σ=σ−2φTφ+A−1,u=σ−2ΣφTt.

Because *p*(*a*, *σ* ^2^∣*t*) ∝ *p*(*t*∣*a*, *σ* ^2^)*p*(*a*)*p*(*σ* ^2^) and *p*(*a*, *σ* ^2^∣*t*) cannot be solved by means of integration, the solution is approximated using the maximum likelihood method, represented by

(20) $$\begin{array}{c} \left(\;a_{MP},\sigma_{MP}^{2}\;\right)=\underset{a,\sigma^{2}}{\arg\max}\;p\left(\;t\;|\;a,\sigma^{2}\;\right). \end{array}$$

The iterative process of *a*_*MP*_ and *σ*_*MP*_^2^ is as follows:

(21) ainew=γiμi2,σ2new=t−φμ2N−∑i=0Nμi,γi=1−ai∑i,i,

where ∑*i*, *i* is *i*th element on the diagonal of Σ and the initial value of *a* and *σ* ^2^ can be determined via the approximation of *a*_*MP*_ and *σ*_*MP*_^2^ by continuously updating using formula (21). After enough iterations, most of *a*_*i*_ will be close to infinity, the value of the corresponding parameters in *w*_*i*_ will be zero, and other *a*_*i*_ values will be close to finite. The resulting corresponding parameters *x*_*i*_ of *a*_*i*_ are now referred to as the relevance vector.

### 2.6. Procedure of the Proposed Method

**Figure 1:**
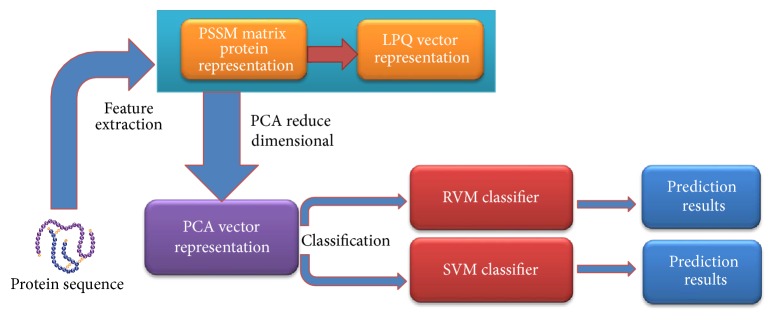
The flow chart of the proposed method.

In the paper, our proposed method contains three steps: feature extraction, dimensionality reduction using PCA, and sample classification. The feature extraction step contains two steps: (1) each protein from the datasets is represented as a PSSM matrix and (2) the PSSM matrix of each protein is expressed as a 256-dimensional vector using the LPQ method. Dimensional reduction of the original feature vector is achieved using the PCA method. Finally, sample classification occurs in two steps: (1) the RVM model is used to carry out classification based on the datasets from* Yeast* and* Human* whose features have been extracted and (2) the SVM model is employed to execute classification on the dataset of* Yeast*. The flow chart of the proposed method is displayed in Figure 1.

### 2.7. Performance Evaluation

To evaluate the feasibility and efficiency of the proposed method, five parameters, the accuracy of prediction (Ac), sensitivity (Sn), specificity (Sp), precision (Pe), and Matthews's correlation coefficient (MCC), were computed. They are represented as follows:

(22) Ac=TP+TNTP+FP+TN+FN,Sn=TPTP+TN,Sp=TNFP+TNPe=TPFP+TP,MCC=TP×TN−FP×FNTP+FN×TN+FP×TP+FP×TN+FN,

where TP, TN, FP, and FN represent true positives, true negatives, false positives, and false negatives, respectively. True positives stand for the number of true interacting pairs correctly predicted. True negatives are the number of true noninteracting pairs predicted correctly. False positives stand for the number of true noninteracting pairs falsely predicted, and false negatives are the number of true interacting pairs falsely predicted to be noninteracting pairs. Moreover, a Receiver Operating Curve (ROC) was created to evaluate the performance of our proposed method.

## 3. Results and Discussion

### 3.1. Performance of the Proposed Method

To avoid the overfitting in the prediction model and to test the reliability of our proposed method, we used 5-fold cross-validation in our experiment. More specifically, the whole dataset was divided into five parts; four parts were employed for training model, and one part was used for testing. Five models were gained from the* Yeast* and* Human* datasets using this method, and each model was executed alone in the experiment. For the sake of ensuring fairness, the related parameters of the RVM model were set up the same for the two different datasets,* Yeast* and* Human*. Here, the Gaussian function was selected as the kernel function with the following parameters: width = 0.6, initapla = 1/*N* ^2^, and beta = 0, where width represents the width of the kernel function, *N* is the number of training samples, and the value of beta was defined as zero, which represents classification. The experimental results of the prediction models of the RVM classifier combined with Local Phase Quantization and the Position Specific Scoring Matrix and Principal Component Analysis based on the protein sequence information from the two datasets are listed in Tables 1 and 2.

Using the proposed method on the* Yeast* dataset, we achieved the results of average accuracy, sensitivity, precision, and MCC of 96.25%, 92.63%, 92.67%, and 87.27%. The standard deviations of these criteria values were 0.95%, 0.55%, 1.40%, and 1.61%, respectively. Similarly, we also obtained good results of average accuracy, sensitivity, precision, and MCC of 97.92%, 99.187%, 96.77%, and 95.95% on the* Human* dataset. The standard deviations of these criteria values were 0.81%, 0.21%, 1.57%, and 1.58%, respectively.

It can be seen from Tables 1 and 2 that the proposed method is accurate, robust, and effective for predicting PPIs. The better performance for predicting PPIs may be attributed to the feature extraction of the proposed method. This approach is novel and effective, and the choice of the classifier is accurate. The proposed feature extraction method contains three data processing steps. First, the PSSM matrix not only describes the order information for the protein sequence but also retains sufficient prior information; thus, it is widely used in other proteomics research. As a result, we converted each protein sequence to a PSSM matrix that contains all the useful information from each protein sequence. Second, because Local Phase Quantization has the advantage of blur invariance in the domain of image feature extraction, information can be effectively captured from the PSSMs using the LPQ method. Finally, while meeting the condition of maintaining the integrity of the information in the PSSM, we reduced the dimensions of each LPQ vector and reduced the influence of noise using Principal Component Analysis. Consequently, the sample information that was extracted using the proposed feature extraction method is very suitable for predicting PPIs.

### 3.2. Comparison with the SVM-Based Method

Although our proposed method achieved reasonably good results on the* Yeast* and* Human* datasets, its performance must be further validated against the state-of-the-art support vector machine (SVM) classifier. More specifically, we compared the classification performances between SVM and RVM model on the* Yeast* dataset using the same feature extraction method. The LIBSVM tool (available at https://www.csie.ntu.edu.tw/~cjlin/libsvmtools/) was employed to carry out classification in SVM. Two corresponding parameters of SVM, *c* and *g*, are optimized using a grid search method. In the experiment, we set *c* = 0.7 and *g* = 0.6 and used a radial basis function as the kernel function.

**Figure 2:**
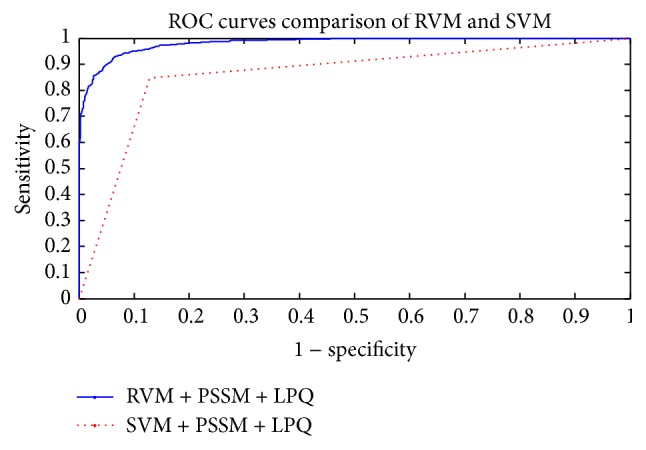
Comparison of ROC curves performed between RVM and SVM on the* Yeast* dataset.

The prediction results of the SVM and RVM methods on* Yeast* dataset are shown in Table 3, and the ROC curves are displayed in Figure 2. From Table 3, the prediction results of the SVM method achieved 85.34% average accuracy, 84.40% average sensitivity, 86.89% average specificity, and 74.97% average MCC, while the prediction results of the RVM method achieved 92.65% average accuracy, 92.63% average sensitivity, 92.67%, average specificity, and 86.40% average MCC. From these results, we can see that the RVM classifier is significantly better than the SVM classifier. In addition, the ROC curves were analyzed in Figure 2, showing that the ROC curve of the RVM classifier is significantly better than that of the SVM classifier. This clearly proves that the RVM classifier of the proposed method is an accurate and robust classifier for predicting PPIs. The increased classification performance of the RVM classifier compared with the SVM classifier can be explained by two reasons: (1) the obvious advantage of RVM is that the computational work of the kernel function is greatly reduced and (2) RVM overcomes the shortcoming of the kernel function being required to satisfy the condition of Mercer. Due to these reasons, the RVM classifier of our proposed method is significantly better than the SVM classifier. At the same time, it has been proven that the proposed method can yield highly accurate PPI predictions.

### 3.3. Comparison with Other Methods

In addition, a number of PPI prediction methods based on protein sequences have been proposed. To prove the effectiveness of our proposed method, we compared the prediction ability of our proposed method, which uses an RVM model combined with a Position Specific Scoring Matrix, Local Phase Quantization, and Principal Component Analysis, with existing methods on* Yeast* and* Human* datasets. It can be seen from Table 4 that the average prediction accuracy of the five different methods is between 75.08% and 89.33% for* Yeast* dataset. The prediction accuracies of these methods are lower than that of the proposed method, which is 92.65%. Similarly, the precision and sensitivity of our proposed method are also superior to those of the other methods. At the same time, Table 5 shows the average prediction accuracy between the six different methods and the proposed method on the* Human* dataset. From Table 5, the prediction accuracies yielded by the other methods are between 89.3% and 96.4%. None of these methods obtains higher prediction accuracy than our proposed method. From Tables 4 and 5, it can be observed that the proposed method yielded obviously better prediction results compared to other existing methods based on ensemble classifiers. All these results prove that the RVM classifier combined with Local Phase Quantization and the Position Specific Scoring Matrix and Principal Component Analysis can improve the prediction accuracy relative to current state-of-the-art methods. Our method improves predictions by using a correct classifier and a novel extraction method that captures the useful evolutionary information.

## 4. Conclusion

Knowledge of PPIs is becoming increasingly more important, which has prompted the development of computational methods. Though many approaches have been developed to solve this problem, the effectiveness and robustness of previous prediction models can still be improved. In this study, we explore a novel method using an RVM classifier combined with Local Phase Quantization and a Position Specific Scoring Matrix. From the experimental results, it can be seen that the prediction accuracy of the proposed method is obviously higher than those of previous methods. It is a very promising and useful support tool for future proteomics research. The main improvements of the proposed method come from adopting an effective feature extraction method that can capture useful evolutionary information. Moreover, the results showed that PCA significantly improves the prediction accuracy by integrating the useful information and reducing the influence of noise. In addition, the experimental results show that the RVM model is suitable for predicting PPIs. In conclusion, the proposed method is an efficient, reliable, and powerful prediction model and can be a useful tool for future proteomics research.

## Figures and Tables

**Table 1:** 5-fold cross-validation results shown by using our proposed method on the Yeast dataset.

Testing set	Ac (%)	Sn (%)	Pe (%)	MCC (%)
1	92.76	92.73	92.79	86.56
2	93.79	93.27	93.41	88.34
3	91.28	92.12	90.43	84.08
4	92.27	92.02	92.50	85.72
5	93.17	93.02	93.32	87.27
Average	92.65 ± 0.95	92.63 ± 0.55	92.67 ± 1.40	86.40 ± 1.61

**Table 2:** 5-fold cross-validation results shown by using our proposed method on the Human dataset.

Testing set	Ac (%)	Sn (%)	Pe (%)	MCC (%)
1	98.10	98.99	97.25	96.27
2	97.67	99.49	96.02	95.45
3	97.37	99.25	95.55	94.87
4	97.24	98.96	95.72	94.63
5	99.26	99.22	99.31	98.54
Average	97.92 ± 0.81	99.18 ± 0.21	96.77 ± 1.57	95.95 ± 1.58

**Table 3:** 5-fold cross-validation results shown by using our proposed method on the Yeast dataset.

Testing set	Ac (%)	Sn (%)	Sp (%)	MCC (%)
SVM + PSSM + LPQ				
1	85.96	84.77	87.13	75.86
2	84.18	82.86	85.43	73.33
3	85.52	84.10	86.97	75.22
4	85.29	84.12	86.47	74.91
5	85.76	86.16	88.45	75.55
Average	85.34 ± 0.69	84.40 ± 1.20	86.89 ± 1.09	74.97 ± 0.98

RVM + PSSM + LPQ				
1	92.76	92.73	92.79	86.56
2	93.79	93.27	93.41	88.34
3	91.28	92.12	90.43	84.08
4	92.27	92.02	92.50	85.72
5	93.17	93.02	93.32	87.27
*Average*	*92.65 ± 0.95 *	*92.63 ± 0.55 *	*92.67 ± 1.40 *	*86.40 ± 1.61*

**Table 4:** Predicting ability of different methods on the Yeast dataset.

Model	Testing set	Ac (%)	Sn (%)	Pe (%)	MCC (%)
Guo et al.'s work [20]	ACC	89.33 ± 2.67	89.93 ± 3.60	88.77 ± 6.16	N/A
	AC	87.36 ± 1.38	87.30 ± 4.68	87.82 ± 4.33	N/A

Zhou et al.'s work [25]	SVM + LD	88.56 ± 0.33	87.37 ± 0.22	89.50 ± 0.60	77.15 ± 0.68

Yang et al.'s work [26]	Cod1	75.08 ± 1.13	75.81 ± 1.20	74.75 ± 1.23	N/A
	Cod2	80.04 ± 1.06	76.77 ± 0.69	82.17 ± 1.35	N/A
	Cod3	80.41 ± 0.47	78.14 ± 0.90	81.66 ± 0.99	N/A
	Cod4	86.15 ± 1.17	81.03 ± 1.74	90.24 ± 1.34	N/A

You et al.'s work [27]	PCA-EELM	87.00 ± 0.29	86.15 ± 0.43	87.59 ± 0.32	77.36 ± 0.44

*The proposed method *	*RVM*	*92.65 ± 0.95 *	*92.63 ± 0.55 *	*92.67 ± 1.40 *	*86.40 ± 1.61*

**Table 5:** Predicting ability of different methods on the Human dataset.

Model	Ac (%)	Sn (%)	Pe (%)	MCC (%)
LDA + RF [28]	96.4	94.2	N/A	92.8
LDA + RoF [28]	95.7	97.6	N/A	91.8
LDA + SVM [28]	90.7	89.7	N/A	81.3
AC + RF [28]	95.5	94.0	N/A	91.4
AC + RoF [28]	95.1	93.3	N/A	91.0
AC + SVM [28]	89.3	94.0	N/A	79.2
*The proposed method *	*97.92*	*99.18*	*96.77*	*95.95*
